# Direct synthesis of ultrafine tetragonal BaTiO_3 _nanoparticles at room temperature

**DOI:** 10.1186/1556-276X-6-466

**Published:** 2011-07-23

**Authors:** Jian Quan Qi, Tao Peng, Yong Ming Hu, Li Sun, Yu Wang, Wan Ping Chen, Long Tu Li, Ce Wen Nan, Helen Lai Wah Chan

**Affiliations:** 1 Department of Materials Sciences and Engineering, Northeastern University at Qinhuangdao Branch, Qinhuangdao, Hebei Province, 066004, Peoples Republic of China; 2 Department of Applied Physics and Materials Research Center, The Hong Kong Polytechnic University, Hong Kong, China; 3 State Key Laboratory of Fine Ceramics and New Processing, Department of Materials Science and Engineering, Tsinghua University, Beijing 100084, China

**Keywords:** BaTiO_3_, nanoparticle, room temperature

## Abstract

A large quantity of ultrafine tetragonal barium titanate (BaTiO_3_) nanoparticles is directly synthesized at room temperature. The crystalline form and grain size are checked by both X-ray diffraction and transmission electron microscopy. The results revealed that the perovskite nanoparticles as fine as 7 nm have been synthesized. The phase transition of the as-prepared nanoparticles is investigated by the temperature-dependent Raman spectrum and shows the similar tendency to that of bulk BaTiO_3 _materials. It is confirmed that the nanoparticles have tetragonal phase at room temperature.

## Introduction

Barium titanate (BaTiO_3_) is widely used for electronic devices in the technological ceramic industry because of its ferroelectric, thermoelectric, and piezoelectric properties when it assumes the tetragonal structure [1]. As such, it can be widely used in capacitors, positive temperature coefficient resistors, dynamic random access memories, electromechanics, and nonlinear optics [2,3]. For the existence of the size effect of ferroelectricity and the potential application of bottom-up assembled novel nanostructures, the synthesis of ultrafine BaTiO_3 _nanoparticles is theoretically and technologically important [4]. Many novel synthesis techniques have been developed for this important material.

The hydrothermal method is one of the most popular approaches to the perovskite nanostructures directly from solution, but the synthesis processes are often conducted at elevated temperatures (typically 100°C to 280°C) and/or under relatively high pressures to improve the crystallinity of the products [5,6]. To avoid high pressure during synthesis, the thermal decompositions of a metal-organic precursor were developed to prepare the nanostructures of BaTiO_3_, SrTiO_3_, BaZrO_3_, and their solid-state solution at around 200°C [4,7-9], but the metal-organic precursors are often expensive. Much effort was done to decrease the synthesis temperature in order to obtain the fine particles with less agglomeration. Direct synthesis from solution (DSS) was developed to prepare perovskite nanoparticles with the particle size of 20 nm to approximately 70 nm, which was operated at 50°C to approximately 100°C and normal pressure [10-12] conveniently by dripping titanium or zirconium alkoxide solution into strong alkaline (i.e., barium hydroxide) solution, but much finer grain size is difficult to obtain and the production efficiency for industry is limited by the low solubility of alkaline earth hydroxides. Recently, barium titanate nanoparticles have been synthesized at room temperature with peptide nanorings as templates [13], or using biosynthesis method [14]. However, it is difficult to enlarge the production scale, the process cannot be controlled facilely, and also the cost of biosynthesis is very high. Above all aqueous systems, cubic phase of BaTiO_3 _are synthesized mostly [6-13]. To obtain the tetragonal phase which has ferroelectricity, annealing at high temperature is necessary and thus grain growth and aggregation are inevitable. To further simplify the process, lower the processing temperature, improve the synthesis efficiency, and acquire much finer grain size and tetragonal phase are important and still rather challenging technically.

In this study, a method is developed to prepare ultrafine tetragonal barium titanate nanoparticles at room temperature. The quantity of the product can be easily enlarged, and the cost is low.

## Experimental procedure

The method is evolved from DSS and is carried out in an enclosed system. The spontaneous reaction of alkali to environmental CO_2 _is avoided, and the content of barium carbonate is suppressed in the final products. The reagents anhydrous Ba(OH)_2 _and tetrabutyl titanate [Ti(OC_4_H_9_)_4_] are adopted as starting raw materials to prepare ultrafine BaTiO_3 _nanoparticles. The titanium solution is obtained by dissolving 34.0 g of Ti(OC_4_H_9_)_4 _into 50.0 ml butanol. The alkali slurry is prepared by ball milling of the mixture of 17.1 g Ba(OH)_2 _and 3.60 g H_2_O in 100 ml butanol for 4 h. The cubage of the milling jar is 250 ml. The titanium solution is added into the alkali slurry in the jar and resealed for another 18-h milling at the rate of 200 rpm; after that, homogenous white slurry is obtained. The white slurry is air-dried, and BaTiO_3 _nanoparticles are synthesized. All of the procedures are carried at room temperature.

The samples are characterized at room temperature by X-ray diffraction (XRD) on a Philips Diffractometer (model: X'Pert-Pro MPD; Philips, Eindhoven, The Netherland) using CuKα radiation (40 kV, 30 mA). The microstructures of the as-prepared powders are observed by transmission electron microscopy (TEM) on a JEOL TEM (model: JSM2010; JEOL Ltd., Tokyo, Japan). The Raman spectra are recorded on an HR800 (Horiba Jobin Yvon, Chilly Mazarin, France) particle analyzer using the laser exciting line of 637, 488, and 325 nm. The rate of measured temperature rise is 15°C/min.

## Results and discussion

A large quantity (23.0 g) of barium titanate nanoparticles is directly synthesized at room temperature. Because ball milling is used as a means of blending, the solubility of barium hydroxide is not a limit during synthesis and thus the synthesis efficiency is improved distinctly. For example, a large quantity of solvents has to be used in a conventional solution method since the solubility of barium hydroxide is low (i.e., 20°C, 3.9 g/100 ml water). In our method, only small quantity of dispersant is needed and the batch of product can be enlarged easily.

**Figure 1:**
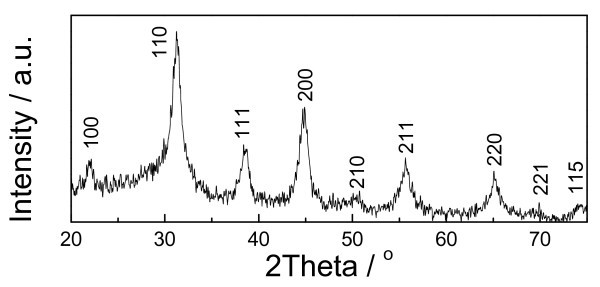
**The XRD profiles of the as-prepared nanoparticles**.

Figure 1 shows the XRD profile of the as-prepared nanoparticles, and the sample has perfectly crystallized perovskite structure. It is believed that the line broadening effect is caused by the fine grains, and the grain size can be estimated as 6.8 nm by Scherrer's equation [15] according to the XRD results.

**Figure 2:**
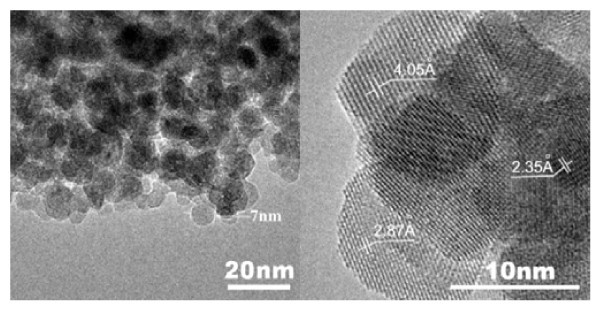
**TEM image**.

The TEM is used for a clear observation in details as shown in Figure 2. The left of Figure 2 is a low-magnitude image, and the average grain size is estimated as approximately 7 nm which also quite agrees with the XRD estimation. The high-magnitude image is shown on the right to show more details of the grain lattice. Regularly arranged patterns can be observed in the darker region of the photo, indicating that the particles under observation are well crystallized. Three patterns of the lattice spacings are observed, such as 4.05, 2.87, and 2.35 Å which match the (100), (110), and (111) perovskite lattice planes, respectively.

**Figure 3:**
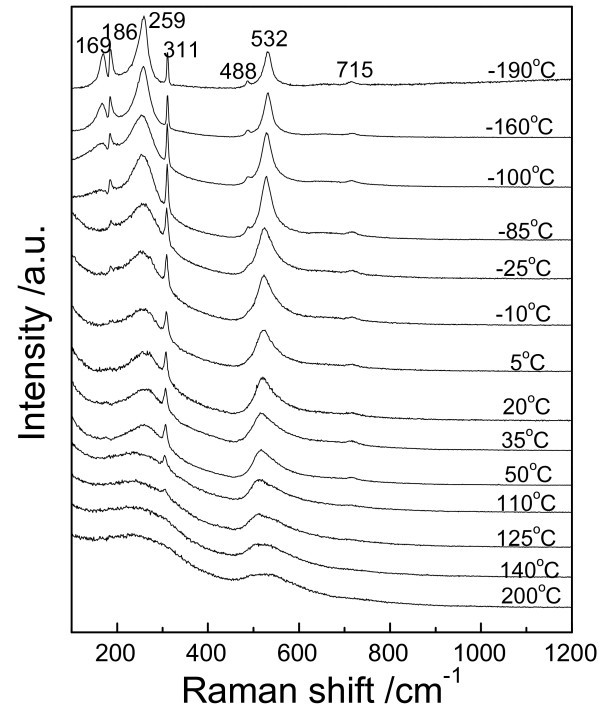
**Temperature-dependent Raman spectra**.

The phase transition of BaTiO_3 _is related to its ferroelectricity because a net displacement of Ti^4+ ^with respect to the O_6_-octahedron in the distortion directions results in the spontaneous polarization in the ferroelectric phases [16]. For the non-ferroelectric cubic phase of BaTiO_3 _nanoparticles that are synthesized in aqueous systems mostly, the study of phase transition is important to check if the nanoparticles have tetragonal phase at room temperature and have ferroelectricity. The tetragonal distortion of BaTiO_3_, *δ *= (*c *- *a*)/*a*, is only 1% in bulk materials and thus is quite difficult to be measured with XRD in nanoparticles for the line broadening effect. The vibrational spectroscopy as Raman spectroscopy is sensitive to the structural transformation, and thus, the local lattice distortions and crystallographic defects at the molecular level can be detected [17]. In our experiment, the sample is found to be pseudo-cubic by XRD. In order to observe the phase transition in BaTiO_3 _nanoparticles, temperature-dependent Raman spectroscopy is used as shown in Figure 3. The detailed phonon assignments for each active modes are: 720 cm^-1^(E(4LO) + A1(3LO)), 515 cm^-1 ^(E(4TO) + A1(3TO)), 305 cm^-1 ^(E(3TO) + E(2LO) + B1), 260 cm^-1 ^(A1(2TO)), and 185 cm^-1 ^(E(2TO) + E(1LO) + A1(1TO) + A1(1LO)) [18,19].

The peak around 310 cm^-1 ^appears below the Curie point and vanishes above the Curie point in BaTiO_3 _ceramics [20], suggesting that the peak at 311 cm^-1 ^(E(3TO) + E(2LO) + B1) in our sample which vanished above 125°C is an intrinsic peak for tetragonal BaTiO_3_. The peaks at 532, 259, and 186 cm^-1 ^are assigned to the fundamental TO mode of A1 symmetry while comprising the main difference in Raman spectra among tetragonal and orthorhombic phases of BaTiO_3 _[21]. The sharp peak at 186 cm^-1 ^(E(2TO) + E(1LO) + A1(1TO) + A1(1LO)) which vanishes above 5°C reveals that it is a feature of orthorhombic phase. A sharp peak 169 cm^-1 ^which appears at very low temperature and vanishes at -90°C shows that it is a characteristic wave band for rhombohedral phase. This peak has been documented in early references as ν3(TO) [22] or A1(TO) [23]. Similar to the peak 169 cm^-1^, the peak 488 cm^-1 ^has been documented as ν1(TO) [22] or E1(TO) [23] and only appears in rhombohedral phase but is rather weak. Although, both peaks at 169 cm^-1 ^and 488 cm^-1 ^appear rarely in recent references, our Raman spectra of 7-nm BaTiO_3 _nanoparticles agree well with early references which have been measured using bulk materials. Overall, the Raman spectroscopy clearly shows that the nanoparticles prepared from our method show the normal phase transition as bulk BaTiO_3 _materials and have tetragonal Raman behavior at room temperature even when the grain size is as small as 7 nm.

The ultrafine tetragonal BaTiO_3 _nanoparticles is synthesiezd in our system. The synthesis mechanism of BaTiO_3 _nanoparticles is believed to undergo two steps [24], hydrolysis of alkoxide to form titanium hydroxide and followed crystallization of BaTiO_3 _nanoparticles by adsorption of Ba^2+^. In our system, the water content is controlled and a suitable dispersent is chosen. Less water (include crystalline water) in the system causes less hydrogen interstitial introduced in the lattice, and thus, tetragonal phase can be achieved. The long alkanol chain of the dispersant, and also less water, depresses the interactions among the nanoparticles or/and dispersents, where ultrafine nanoparticles with less aggregation can be obtained. The more details of crystalline mechanism will be studied further. The ferroelectricity of the composite with the polymer and the ultrafine BaTiO_3 _nanoparticles will be done in the future.

## Conclusion

A large quantity of tetragonal BaTiO_3 _nanoparticles as fine as 7 nm was directly synthesized at room temperature. The synthesis efficiency improved distinctly, and the batch processing could be scaled up easily because large quantity of solvents was not necessary in the method. Both XRD and TEM results revealed that the as-prepared nanoparticles had perfect crystallized perovskite phase with ultrafine grain size. Temperature-dependent Raman spectrum shows that the nanoparticles prepared from our method have the normal phase transition as bulk BaTiO_3 _materials and have tetragonal phase at room temperature even when the grain size is as small as 7 nm.

## Competing interests

The authors declare that they have no competing interests.

## Authors' contributions

JQQ participated in the design of the study, explained the XRD and TEM images and contributed in the writing of the manuscript. TP participated in the synthesis of the samples. YMH measured and explained TEM. LS measured and explained XRD. YW measured Raman spectra. WPC explained Raman spectra. LTL participated in disscuss of the study. CWN participated in disscuss of the results. HLWC participated in revision of the manuscript and discuss of the results. All authors read and approved the final manuscript.
